# Oxidative stress is associated with markers of renal dysfunction in children aged 6-9 years old in a South African population

**DOI:** 10.11604/pamj.2022.42.35.26443

**Published:** 2022-05-13

**Authors:** Edna Ngoakoana Matjuda, Godwill Azeh Engwa, Muhau Muhulo Mungamba, Constance Rufaro Sewani-Rusike, Benedicta Ngwechi Nkeh-Chungag

**Affiliations:** 1 Department of Human Biology, Faculty of Health Sciences, Walter Sisulu University PBX1, 5117, Mthatha, South Africa,; 2 Department of Biological and Environmental Sciences, Faculty of Health Sciences, Walter Sisulu University PBX1, 5117, Mthatha, South Africa

**Keywords:** Oxidative stress, albuminuria, renal dysfunction, cardiovascular diseases

## Abstract

**Introduction:**

although studies have shown a relationship between albuminuria and oxidative stress in adults, limited information on the subject is available in children. The aim of this study was to assess the relationship between oxidative stress and albuminuria in South African children of African ancestry.

**Methods:**

a cross-sectional study involving 6-9 years old children in randomly selected rural and urban schools of the Eastern Cape Province of South Africa was conducted. Anthropometric measurements were done and urine samples were collected for the determination of titres of albumin, creatinine, 8-hydroxy-2-deoxy-guanosine (8-OHdG) and thiobarbituric acid reactive substances (TBARS). The urinary albumin to creatinine ratio (ACR) was calculated and used to determine albuminuria.

**Results:**

creatinine and 8-OHdG were significantly higher (p<0.05) in urban children than in rural children while albumin, ACR and TBARS were significantly higher (p<0.05) in rural compared to urban children. The prevalence of albuminuria was 14.05% of which microalbuminuria was 8.83% while macroalbuminuria was 5.22%. Albuminuria was higher in rural children than their urban counterparts and was more prevalent in females. TBARS was positively (p<0.05) associated with creatinine and albumin in the cohort as well as in females and urban children while 8-OHdG was positively associated with albumin in the cohort.

**Conclusion:**

findings of this study showed that oxidative stress was associated with markers of renal dysfunction with a 14% prevalence of albuminuria observed in South African children of African ancestry.

## Introduction

Albuminuria is a preclinical marker of a public health concern as it predicts the early development of cardiovascular diseases (CVDs) and renal disease in adults [1]. It is a marker of glomerular damage that predicts progressive renal failure in conditions such as diabetes mellitus which is also associated with hyperperfusion and hyperfiltration [2]. Microalbuminuria is defined as an abnormal or supranormal urinary excretion of albumin in the absence of clinical proteinuria. However, prolonged period of microalbuminuria is proceeded by persistent proteinuria which is subsequently followed by chronic renal disease [3]. Since the root of CVDs and chronic renal disease is tracked back to childhood, the assessment of microalbuminuria has become relevant in child and adolescent clinical care. Although there have been large population-based studies defining normal levels and correlates of albumin excretion in adults [1], there have been fewer studies in children. More so, most of these studies have based their measurements on overnight albumin excretion rate (AER) as opposed to urinary albumin/creatinine ratios (ACRs) [4], which is reliably used to measure microalbuminuria, a recognized early marker of renal dysfunction [5]. Considering the clinical concern of microalbuminuria as a marker of renal dysfunction, identification of its risk factors may allow earlier intervention to prevent renal complications.

Albuminuria has been associated with risk of CVD, chronic kidney disease (CKD), diabetes, hypertension [6,7] and sickle cell anaemia [8]. Also, several risk factors such as dyslipidaemia, inflammation, increased renin-angiotensin system markers, oxidative stress, elevated levels of homocysteine, uraemic toxins and thrombogenic factors, smoking, and increasing age have been associated with CKD as well as albuminuria [6, 9, 10]. There is evidence that some newly identified biomarkers in CKD are related to oxidative stress [11, 12]. Oxidative stress, a condition which is commonly observed in most diseases occurs as a result of excessively high level of free radicals which overwhelms the antioxidant system of the body. Oxidative stress is involved in the pathogenesis of many diseases and can induce cellular injury or organ dysfunctions [13, 14]. Reactive oxygen species (ROS) which cause oxidative stress may result from intrinsic metabolic or physiological processes but may also originate from external sources from the environment such as air pollution, smoking etc. which are dependent on human activity. The variation of human activities between rural and urban settings may affect the generation of ROS and the development of oxidative stress [15]. Besides the fact that ROS can lead to oxidative damage of proteins, lipids, and nucleic acids, it is also known to disrupt cellular function damaging tissues and organs in the body including the kidney [16-18]. Evaluation of oxidative stress can be done by assessing certain biomarkers which are modifiable products that result from the interaction of ROS with biomolecules such as lipids, proteins, nucleic acids etc. Oxidative damage of lipids during lipid peroxidation results in the formation of malondialdehyde and thiobarbituric acid reactive substances (TBARS) which are useful indicators for assessing lipid oxidative damages [19]. Also, 8-hydroxy-2´-deoxyguanosine (8-OHdG) is a maker for Deoxyribonucleic acid (DNA) damage as it is increased following the interaction between oxidative compounds with nucleic acids [20].

The progression of CKD to advanced stages is associated with a significant increase in the generation of ROS [21]. Markers of oxidative RNA and DNA damage have been related to albuminuria even among apparently normal individuals [22]. Accordingly, urinary levels of 8-oxo-7, 8-dihydroguanosine (8-oxoGuo) were shown to be independently associated with incident low-grade albuminuria in individuals with renal disease, diabetes or CVD [23]. Though there is evidence of the association between oxidative stress markers and albuminuria with deteriorating renal function in adults [1, 24, 25], limited information of such relationship is available in children. Thus, this study was aimed to assess the relationship between oxidative stress and albuminuria in South African children of African ancestry.

## Methods

**Study population and design:** this was a cross-sectional study that involved primary school children of African Ancestry aged 6-9 years from rural and urban areas of the Eastern Cape Province of South Africa. The children were recruited from primary schools in Libode, a rural area and from Mthatha and East London which are urban areas. The sample size of the study was calculated using the formula:


$$n=\frac{Z^{2}p(1-p)}{d^{2}}$$

Where n=sample size, Z^2^ = confidence interval (1.96), p=estimate population size (27%), and d=desired precision (0.05). From the calculation, sample size (n) = [(1.96)^2^*0.27(1-0.27)]/(0.052)^2^=303. Thus, a total sample size of three hundred and three (303) children was obtained. Hence, a minimum of 150 children were recruited from each of the sites (urban and rural areas).

**Ethical consideration:** the study was conducted in accordance with the guidelines of the Helsinki Declaration (2008 reviewed version) [26] as well as local and national regulations in South Africa [27]. Ethical approval was obtained from Walter Sisulu University Health Sciences Ethics Committee (Ref No: 112/2018). After careful explanation of the purpose and aim of the study, written informed consent was obtained from the parents/legal guardians of the children before enrolment into the study. The study adhered to the standards of reporting and was in accordance with the National Data Protection Acts as the identity of the participants was kept confidential. There were no important changes to the methods after study commencement.

**Inclusion/exclusion criteria:** children of African ancestry aged 6-9 years, who were free from cardiovascular and renal diseases, were recruited for the study. Ill and physically challenged children having any self-reported comorbidity or CVDs as well as children who are not of African ancestry were excluded from the study.

**Data collection and biochemical analysis:** participants´ height and weight were measured and used to calculate the body mass index (BMI) as weight (kg)/ height^2^ (m^2^). Urine was collected from all participants in sterile tubes and was used to quantify the following biochemical parameters. Creatinine was quantified using the Roche Cobas 6000 analyser while albumin, and 8-Hydroxy-2´-deoxyguanosine (8-OHdG) were determined using ELISA kits as per manufacturers´ protocol. Urinary albumin to creatinine ratio (ACR) was calculated and classified as normal: <3 mg/mmol, 3 ≥ACR ≤ 30: moderately increased or microalbuminuria, and ACR >30: severely increased and macroalbuminuria [28]. Lipid peroxidation assay was performed based on the quantification of thiobarbituric acid reactive substances (TBARs) as described by Mallick and colleagues [29].

**Statistical analysis:** stata MP version 14.1 was used for data analyses. Data were expressed as mean± confidence interval. Analysis of variance was used to compare the mean differences of study parameters based on location and sex. Pearson correlation was used to evaluate the relationship between renal function markers and oxidative stress indices. A 95% confidence interval was employed and a p-value ≤ 0.05 was considered significant.

**Funding:** this study was funded by the South African National Research Foundation NRF-CPRR, Grant No. 106066 to Benedicta Ngwechi Nkeh-Chungag.

## Results

**General characteristics of study participants:** three hundred and six (306) children were recruited for the study which included 152 children from rural areas and 154 from urban areas. Among the 152 rural children, there were 83 females and 69 males while among the urban 154 children 88 were females and 66 were males. The ages, weight and height as well as the BMI of children were not significantly different (p = 0.198) between rural and urban areas. Thus, the study population matched for age, weight and height in the urban and rural areas. Albumin, ACR and TBARS were higher (p<0.001) in rural compared to urban children while creatinine and 8-OHdG were higher (p<0.001) in urban compared to rural children as summarised in Table 1.

**Table 1:** baseline characteristics of participating children by sex and location

Variables	Rural (95% CI)		Urban (95% CI)		p-value
	Girls	Boys	Girls	Boys	
	N=83	N=69	N=88	N=66	
Age (y)	7.91(7.65-8.17)	7.88(7.56-8.19)	8.34(8.08-6.67)	8.11(7.72-8.49)	0.320
HT (m)	1.25 (1.23-1.27)	1.27 (1.24-1.29)	1.29 (1.25-1.34)	1.28 (1.25-1.30)	0.198
WT(kg)	25.44(24.40-26.40)	27.46(25.11-29.82)	28.61(26.60-30.61)	28.10(26.03-30.17)	0.137
BMI (m^2^/kg)	16.4(15.8-16.9)	16.8(15.8-17.9)	17.2(16.4-18.0)	17.1(16.0-18.2)	0.276
Creatinine (mmol/L)	7.17(6.16-8.11)	8.65(6.52-10.78)	10.79(9.03-12.57)	8.46(6.53-10.37)	<0.001
Albumin (mg/L)	47.06(-7.48-101.71)	38.66(-9.75-87.07)	41.78(-9.09-92.65)	5.02(2.65-7.38)	<0.001
ACR (mg/mmol)	6.16(-0.01-12.33)	3.40(-0.90-7.71)	4.17(-1.51-9.85)	0.58(0.38-0.77)	<0.001
TBARS (μM)	0.08(0.07-0.08)	0.09(0.05-0.12)	0.08(0.06-0.09)	0.07(0.05-0.08)	<0.001
8-OHdG (ng/ml)	61.64(57.40-65.88)	66.66(58.34-74.97)	64.92(60.24-69.59)	65.53(59.55-71.51)	<0.001

Values are expressed as mean (min CI-max CI); CI: Confidence interval; N=Number of children; Age(y) = Age in years; HT= Height; WT=Weight; BMI: Body mass index; ACR= Albumin to creatinine ratio; TBARS = Thiorbarbituric acid reactive substance; 8-OHdG 8-hydroxyl-deoxy-guanosine

**Prevalence of albuminuria in children:** a total of 249 urine samples were analysed for ACR. The overall prevalence of albuminuria was 14.05% (35/249) of which microalbuminuria (moderately increased ACR) was 8.83% (22/249) while macroalbuminuria (severely increased ACR) was 5.22% (13/249). Albuminuria was generally higher in rural children than their urban counterparts. More so, the prevalence of albuminuria was higher in females than males. Rural girls had slightly moderately increased ACR than rural boys. Moderately increased ACR was not observed in urban boys. However, urban girls had moderately increased ACR. Moderately increased ACR was higher in rural girls than boys. Rural girls had a higher prevalence of severely increased ACR than rural boys (Table 2).

**Table 2:** albumin to creatinine ratio values of children by sex and location

ACR	Cohort (%)	Rural (%)	Urban (%)	Rural N (%)		Urban N (%)	
				Girls	Boys	Girls	Boys
**Normal**	128 (86.0)	115 (46.6)	103 (41.4)	67 (26.9)	48 (19.3)	51 (20.4)	52 (20.9)
**AU**	35 (14.0)	24 (9.6)	11 (4.4)	16 (6.4)	8 (3.4)	10 (4.0)	1 (0.4)
**MAU**	22 (8.8)	17(6.8)	5 (2.0)	10 (4.0)	7 (2.8)	5 (2.0)	0 (0.0)
**MAAU**	13 (5.2)	7 (2.8)	6 (2.4)	6 (2.4)	1 (0.4)	5 (2.0)	1 (0.4)

N= Number of children; %= Prevalence; ACR: Albumin to creatinine ratio; AU: Albuminuria; MAU: Microalbuminuria; MAAU: Macroalbuminuria

**Relationship between oxidative stress and renal function markers based on location:** creatinine and albumin positively correlated (p<0.01) with TBARS in the cohort and in urban children while 8-OHdG positively correlated (p<0.05) only with albumin in the cohort (Table 3A). Age-adjusted linear regression of a fitted model (F=4.72; p=0.003) for the relationship of TBARS with renal function markers showed increased TBARS to predict increased creatinine in the cohort (R2= 0.079, Adj.R2= 0.062; p=0.008). Also, increased TBARS predicted increased albumin in urban children (p<0.05). However, increased 8-OHdG did not predict any renal function markers (Table 3B).

**Table 3:** relationship correlation between oxidative stress and renal function markers in urban and rural children

3A:			TBARS			8-OHdG	
**Correlation**	r	**Cohort**	**Rural**	**Urban**	**Cohort**	**Rural**	**Urban**
	Creatinine	0.246**	0.143	0.352**	0.091	0.135	0.031
	Albumin	0.165*	0.175	0.243*	0.159*	0.008	-0.029
	ACR	0.101	0.158	0.067	-0.005	-0.007	-0.060
**3B:**			**TBARS**			**8-OHdG**	
**Regression**	β	**Cohort**	**Rural**	**Urban**	**Cohort**	**Rural**	**Urban**
	Creatinine	0.211**	0.134	-0.354***	0.088	0.138	0.017
	Albumin	0.235	0.077	4.886***	0.015	-0.021	0.059
	ACR	-0.115	0.091	-4.654***	-0.039	0.011	-0.109

β: Regression coefficient; r: correlation coefficient; *p<0.05, **p<0.01, ***p<0.001

**Relationship between oxidative stress and renal function markers based on sex:** TBARS positively (p<0.05) correlated with creatinine and albumin in the cohort and in females while 8-OHdG positively correlated with albumin in the cohort (Table 4A). In females, a fitted model (f=6.61; p<0.001) showed increased TBARS to predict increased creatinine (R2= 0.18, Adj.R2= 0.153; p<0.001) while an unfitted model in males ((F=2.27; p=0.088) showed increased TBARS to predict increased albumin and reduced ACR (R2= 0.087, Adj.R2= 0.049; p<0.05) (Table 4B).

**Table 4:** relationship correlation between oxidative stress and renal function markers by gender

4A:		TBARS		8-OHdG	
**Correlation**	r	**Female**	**Male**	**Female**	**Male**
	Creatinine	0.406***	0.096	0.075	0.084
	Albumin	0.222*	-0.030	-0.004	0.005
	ACR	0.150	-0.074	-0.030	-0.015
**4B:**		**TBARS**		**8-OHdG**	
**Regression**	β	**Female**	**Male**	**Female**	**Male**
	Creatinine	0.381***	-0.095	0.073	0.076
	Albumin	0.082	2.115*	0.019	0.056
	ACR	0.050	-2.162*	-0.052	-0.063

β: Regression coefficient; r: correlation coefficient; *p<0.05, ***p<0.001

## Discussion

This study showed an association between oxidative stress and markers of renal dysfunction in children of African origin in South Africa. Albuminuria is widely known to predict renal diseases and CVD in healthy individuals as well as those with hypertension and diabetes [30, 31]. These diseases which are generally more prevalent in adults are also being observed in children and adolescents. Albuminuria and creatininuria are well-established markers of kidney damage [5, 31]. Furthermore, albuminuria which is reliably expressed by urinary albumin to creatinine ratio [32], is widely recognized as an early marker for renal dysfunction [5]. The measurement of these markers has increasingly become of clinical interest in children since the roots of CKD and related diseases in adults are traced back to childhood. The prevalence of albuminuria which is defined by an ACR above 3 mg/mmol in this study was 14.05% and was higher in rural children than their urban counterparts, especially in girls. This prevalence was comparable to the 10-15% prevalence reported by Mogensen and colleagues involving a general population of all age groups [33]. On the other hand, Jones and colleagues reported a lower prevalence of 7.8% in an American study involving children, adolescents, and adults [34]. Our study therefore, reports on a prevalence value midway between values obtained in these two studies.

There is abundant of clinical and experimental evidence that suggests that oxidative stress may be associated with renal dysfunction and thus, play a key role in the pathogenesis of CKD [35,36]. More so, pro-oxidants which are responsible for oxidative stress are known to originate from intrinsic respiratory and metabolic processes in the body [36] but also from environmental sources such as air pollution, smoking etc. [37]. Thus, oxidative stress may be affected by the variation of activities between urban and rural areas. Our findings showed that oxidative stress markers were mostly elevated in rural children. This suggests that rural children are more prone to oxidative stress than their urban counterparts. This finding is in contrast with previous studies that have shown oxidative stress to be elevated in urban than rural areas. A study showed that oxidative stress level was 31% higher for adolescents living in Chivasso (urban site) than for those living in Casalborgone (rural site) [38]. More so, another study showed elderly individuals in urban areas to have more oxidative stress and a higher risk of developing cognitive impairment than those in a rural environment [39]. The high level of oxidative stress in rural children may be a result of the increased air population due to the presence of untarred roads and use of solid fuels sources.

Though previous studies have established the associations between oxidative stress and renal dysfunction based on albuminuria in adults [6, 35], there is paucity of data on this relationship in children. Assessment of the relationship between oxidative stress and renal dysfunction markers in this study showed 8-OHdG was positively associated with albumin. Also, TBARS was positively associated with creatinine as well as albumin. Further, increased TBARS was shown to predict elevated creatinine in the cohort as well as albumin and ACR in males. These findings suggest that oxidative stress is associated with albuminuria, an early sign of renal damage. Previous studies have shown oxidative stress to be associated with microalbuminuria in adults [24, 25], as well as with CKD and other kidney-related diseases in children [13, 40]. Al-Biltagi and colleagues [41] showed oxidative stress to be significantly present in children with end-stage renal disease.

Oxidative stress which is characterised by high levels of pro-oxidants that overwhelm the antioxidant defence system may attack biomolecules such as lipids, proteins and nucleic acids [42] in the tissues of the kidney thereby damaging the kidney. This malfunctioning kidney may manifest as increased urinary albumin. It has also been suggested that oxidative stress may promote inflammation in the kidney which in turn damages the kidney further [43]. The effects of oxidative stress on renal function markers in this study were more prominent with TBARS than 8-OHdG suggesting that cell membrane damage due to lipid peroxidation may be more obvious than oxidative damage of DNA by 8-OHdG or may occur earlier. The association of oxidative stress with markers of renal dysfunction observed especially in urban children in this study is an important public health concern.

Few studies have addressed the impact of oxidative on the renal function of children, although there is sufficient information on the subject in the adult population. This study is among the few studies that have shown the contribution of oxidative stress on renal dysfunction in a South African children population of African origin. Although relative suitable sample size was used suggesting the reliability of the study, the findings of this study may be limiting to generalization as only a single ethnic population; children of African ancestry were considered for the study. Limited parameters were used to assess oxidative stress and renal dysfunction, and therefore, the findings of this study may be limited to these parameters assessed. More so, this was a cross-sectional study and therefore an association between oxidative stress and renal dysfunction markers may not suggest the cause of microalbuminuria in children. A more robust longitudinal study model will be needed to better assess the implication of oxidative stress on the renal function of children in this population.

## Conclusion

This study showed a 14% prevalence of albuminuria and oxidative stress was associated with markers of renal dysfunction in South African children of African ancestry. More so, the relationship between oxidative stress and renal function markers were more prevalent in urban than rural children. The presence of microalbuminuria, an early marker for renal disease, which may have been influenced by oxidative stress calls for public health concern.

### What is known about this topic

- It has been reported that there exists a relationship between albuminuria and oxidative stress in adults;
- However, there is limited information available for children.

### What this study adds

- This study has revealed that oxidative stress is associated with markers of renal dysfunction with an elevated prevalence of albuminuria in South African children of African ancestry.
